# The emerging role of exercise preconditioning in preventing skeletal muscle atrophy

**DOI:** 10.3389/fphys.2025.1559594

**Published:** 2025-03-26

**Authors:** Xu Zhou, Shiming Li, Lu Wang, Jun Wang, Peng Zhang, Xiaoping Chen

**Affiliations:** ^1^ Department of Exercise Physiology, Beijing Sport University, Beijing, China; ^2^ National Key Laboratory of Human Factors Engineering, China Astronaut Research and Training Center, Beijing, China; ^3^ College of Chemistry and Life Science, Beijing University of Technology, Beijing, China; ^4^ National Key Laboratory of Space Medicine, China Astronaut Research and Training Center, Beijing, China

**Keywords:** exercise preconditioning, muscular atrophy, disuse atrophy, sarcopenia, mitochondrial dysfunction

## Abstract

Skeletal muscle atrophy, characterized by the loss of muscle mass and function, can result from disuse, aging, disease, drug. Exercise preconditioning—a form of exercise training performed before these harmful threats—induces notable remodeling and extensive biochemical adaptations in skeletal muscle, creating a protective phenotype in muscle fibers, and thus serving as an effective intervention for preventing skeletal muscle atrophy. Here, we review the current understanding relating to how exercise preconditioning protects skeletal muscle from damage caused by inactivity, sarcopenia, disease, or pharmacological intervention, with an emphasis on the cellular mechanisms involved. Key mechanisms highlighted as making a significant contribution to the protective effects of exercise on skeletal muscle fibers include mitochondria; the expression of cytoprotective proteins such as HSP72, SOD2, SESN2, PGC-1α and AMPK; and the regulation of oxidative stress. These findings underscore the potential of exercise preconditioning as a non-pharmacological intervention for preserving muscle mass and function, as well as preventing muscular atrophy, ultimately improving the quality of life for at-risk populations.

## 1 Introduction

Skeletal muscle fitness and plasticity are important determinants of human health. Skeletal muscle accounts for approximately 35%–40% of total body weight and plays a pivotal role in locomotion, metabolism, respiration, thermogenesis, and endocrine regulation (Maestroni et al., 2020). Furthermore, skeletal muscle demonstrates a remarkable capacity to rapidly adapt to both physiological and pathological stimuli, with this high plasticity enabling functional and structural remodeling (McGee and Hargreaves, 2020). However, under detrimental circumstances such as immobility (e.g., bed rest, spaceflight, denervation), aging, pharmacological intervention [e.g., doxorubicin (DOX) and dexamethasone (DEX)], or pathological conditions, muscle protein synthesis is suppressed, while protein degradation is accelerated, ultimately leading to muscle atrophy (Yin et al., 2021). This not only affects the quality of life but also increases the risk of falls, fractures, frailty, disability, and mortality (Nunes et al., 2022; Mirzoev, 2020).

In contrast, mechanical load from physical exercise serves as a potent stimulus for eliciting adaptive responses at the physiological level. In response to exercise-induced stimuli, a variety of genes and proteins are activated, which induces changes in organelles and structures within skeletal muscle. These molecules, integral to the body’s regulatory systems, mediate acute responses at multiple levels and contribute to long-term adaptive responses to repeated exercise (McGee and Hargreaves, 2020; Egan and Sharples, 2023). These adaptive responses protect muscle fibers even after cessation of exercise, safeguarding them from disuse, aging, drug-induced damage, or pathological insult (Al-Nassan and Fujino, 2018; Kavazis et al., 1985; Theilen et al., 1985; Yoshihara et al., 2019; Smuder et al., 1985a; Smuder et al., 1985b). The phenotypic changes in muscle induced by exercise that confer resistance to atrophy are commonly referred to as “exercise preconditioning” (Powers et al., 2020a). Notably, even brief interventions, such as 2 weeks of aerobic exercise preconditioning, have been demonstrated to serve as effective preventive strategies against disuse and DOX-induced muscle atrophy (Kavazis et al., 1985; Smuder et al., 1985a; Smuder et al., 1985b; Powers et al., 2020a). Similarly, 11 weeks of incremental resistance training effectively counteracted DEX-induced muscle atrophy (Yang et al., 2023), while exercise preconditioning has been reported to ameliorate sarcopenic symptoms and improve mobility in older adults (McKendry et al., 2020; Gries et al., 1985; Dodds et al., 2013; Liu S. et al., 2021).

In this review, we outline the protective effects of exercise preconditioning on skeletal muscle fibers against a variety of harmful events, including disuse, aging, cachexia and pharmacological intervention. Furthermore, we explore the mechanisms through which exercise preconditioning fosters a protective phenotype in skeletal muscle fibers that counteracts these deleterious factors.

## 2 Skeletal muscle atrophy

### 2.1 Disuse atrophy

**FIGURE 1:**
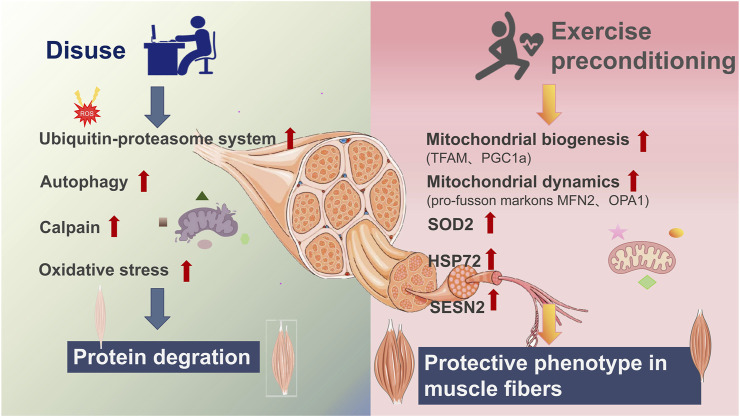
Exercise preconditioning prevents disuse atrophy. Disuse due to conditions such as bed rest or exposure to microgravity leads to skeletal muscle atrophy by promoting protein degradation through the ubiquitin-proteasome system, autophagy, and calpain activation. This is accompanied by increased oxidative stress [generation of reactive oxygen species (ROS)] and mitochondrial dysfunction, resulting in the loss of cytoprotective proteins and the impairment of muscle quality. Exercise preconditioning mitigates against these detrimental effects by inducing a protective muscle phenotype, characterized by enhanced mitochondrial biogenesis (TFAM, PGC-1α), improved mitochondrial dynamics (pro-fusion markers MFN2, OPA1), elevated antioxidant defenses (SOD2), and increased expression of cytoprotective proteins (HSP72, SESN2).

Disuse-induced muscle atrophy, characterized by muscle mass loss and a slow-to-fast fiber-type shift, is commonly induced by prolonged mechanical unloading due to bed rest, limb immobilization, or microgravity exposure (Sergeeva et al., 2024; Ciciliot et al., 2013). During disuse atrophy, mitochondrial dysfunction is a key factor contributing to muscle decline. Mitochondria exhibit reduced maximal respiration, increased reactive oxygen species (ROS) production (Qualls et al., 2021; Kubat et al., 2023; Powers et al., 2012; Long et al., 2025), and higher electron leak, which disrupt metabolic homeostasis and exacerbate muscle atrophy. Additionally, the expression of cytoprotective proteins, such as heat shock proteins, is downregulated (Powers et al., 2020a). These changes collectively lead to muscle weakness and impaired function (Figure 1).

#### 2.1.1 Mitochondria

##### 2.1.1.1 Mitochondria dysfunction

Mitochondria are essential organelles that play a crucial role in regulating the metabolic status of skeletal muscle by orchestrating ATP production and key metabolic pathways (Gan et al., 2018). In response to diverse physiological and pathophysiological stresses, skeletal muscle mitochondria undergo adaptive reprogramming to ensure that ATP production aligns with the energy demands of the muscle, especially muscle contraction. Mitochondrial dysfunction caused by disuse, is characterized by reduced energy production and mitochondrial content, altered mitochondrial morphology, increased oxidative stress, and activation of mitophagy (Qualls et al., 2021; Kubat et al., 2023; Powers et al., 2012; Long et al., 2025). Notably, exercise mitigates disuse-induced mitochondrial dysfunction by promoting mitochondrial network remodeling, specifically through enhanced mitochondrial biogenesis and optimized dynamic regulation (Theilen et al., 1985; Nilsson and Tarnopolsky, 2019; Smuder et al., 1985c; Brocca et al., 2021; Smith et al., 2023; Sandri et al., 2006). Understanding the molecular mechanisms underlying exercise-induced mitochondrial adaptations is critical to optimizing strategies for preserving muscle function and preventing disuse-related atrophy.

Mitochondrial biogenesis is an adaptive regulatory mechanism by increasing mitochondrial number and size to meet heightened energy demands or respond to specific cellular signals (Liu et al., 2025). This process is governed by key regulators, including peroxisome proliferator-activated receptor γ co‐activator 1α and mitochondrial transcription factor A (TFAM), which directly regulates mtDNA transcription. Disuse results in a substantial reduction of mitochondrial content in skeletal muscle, accompanied by decreased expression of key regulators, including PGC-1α, TFAM and Cytochrome c oxidase subunit IV, nuclear respiratory factors 1/2 (NRF1/2), estrogen-related receptor alpha (ERRα), Adenosine monophosphate-activated protein kinase (AMPK) (Gan et al., 2018; Kou et al., 2018; Teodoro et al., 2014; Theilen et al., 2019; Adhihetty et al., 2007; Hyatt and Powers, 2021). PGC-1α (30) or TFAM overexpression (Theilen et al., 2019) mitigates disuse-induced muscle damage, underscoring the therapeutic potential of enhancing mitochondrial biogenesis. As expected, 2-week exercise training prevents hindlimb unloading-induced atrophy in soleus and gastrocnemius muscles by elevating TFAM levels (Theilen et al., 2019; Smuder et al., 2019). Similarly, Theilen et al. (1985) demonstrated that a 2-week aerobic exercise preconditioning reduced oxidative stress by elevating PGC-1α (protein) and TFAM (gene) levels in limb muscles. However, in a mechanical ventilation (MV) model, exercise preconditioning prevented diaphragm atrophy without altering PGC-1α levels (Smuder et al., 1985c). This discrepancy suggests tissue-specific differences (e.g., limb *versus* respiratory muscles) or the involvement of unrecognized regulatory nodes in mitochondrial biogenesis. Future studies should elucidate the roles of other mitochondrial biogenesis markers—such as NRF1/2, TFAM, ERRα and AMPK—in mediating these protective effects across various disuse paradigms.

Mitochondria are dynamic organelles that undergo mitochondrial dynamics—a process encompassing ​fusion, fission, autophagy, and translocation—to maintain mitochondrial function, cellular energy distribution, and signaling (Chan, 2020). However, disuse shifts this balance toward excessive fission, ​a change linked to increased oxidative stress and accelerated muscle protein breakdown (Long et al., 2025; Romanello and Sandri, 2015). Endurance training counteracts this imbalance by upregulating mitochondrial fusion proteins mitofusin 1/2 (MFN1/2) and modulating the fission mediator dynamin-related protein 1 (DRP1), thereby preserving muscle integrity (Hood et al., 2019). Brocca et al. (2021) reported that 7 days of exercise preconditioning prevented hindlimb unloading-induced atrophy, which was associated with the normalization of mitochondrial dynamics during the early stages (within 3 days), as well as the continued alleviation of atrophy (up to 7 days). This finding suggests that exercise preconditioning helps to preserve mitochondrial dynamics, contributing to its early protective effect against disuse-induced atrophy (Brocca et al., 2021).

##### 2.1.1.2 Antioxidants

Disuse-induced muscle atrophy and oxidative stress are closely associated, with each condition exacerbating the other. Elevated ROS levels activate multiple proteolytic systems, while simultaneously inhibiting protein synthesis and weakening the antioxidant defense system, a cascade that accelerates muscle atrophy (Powers et al., 2020b). Treatment with specific antioxidants has been shown to protect skeletal muscle from disuse-induced oxidative stress and subsequent atrophy (Powers et al., 2020a), suggesting that enhancing mitochondrial antioxidant capacity is an effective strategy for mitigating oxidative stress and preventing muscle atrophy. Moderate exercise could effectively enhance antioxidant enzyme activities so that to reduce oxidative stress (Mason et al., 2020), endurance exercise preconditioning has also been demonstrated to protect against disuse-induced oxidative stress, proteolysis, and muscle atrophy (Theilen et al., 1985; Smuder et al., 1985c; Smuder et al., 2019; Morton et al., 2019). 2 weeks of treadmill training (30 m/min, 0% grade, 60 min/day) can significantly prevent MV-induced disuse atrophy by increasing the expression of the antioxidant enzymes superoxide dismutase one and superoxide dismutase 2 (SOD2) within the diaphragm muscle (Smuder et al., 1985c; Smuder et al., 2019; Morton et al., 2019). When exercise-induced increases in diaphragmatic SOD2 levels are prevented, the protective effect of exercise against MV-mediated diaphragmatic atrophy is lost, underscoring the essential role of SOD2 in mediating the benefits of exercise (Morton et al., 2019). However, the overexpression of SOD2 alone could not fully prevent diaphragm atrophy, suggesting that additional factors or pathways are necessary to achieve comprehensive protection.

#### 2.1.2 HSP72

Heat shock protein 72 (HSP72), a member of the heat shock protein family, is upregulated in response to diverse stress stimuli, including heat shock, oxidative stress and mechanical loading. This protein, which functions as a molecular chaperone, has a pivotal role in cellular stress responses, facilitating the proper folding of oxidized proteins, protecting mitochondria from apoptotic signaling, and reducing proteolysis through the inhibition of key proteolytic systems (Wiggs, 2015). The levels of HSP72 are also closely associated with the regulation of muscle mass, and a reduction in its levels is strongly associated to skeletal muscle atrophy. In conditions such as immobilization, tail suspension, or denervation, HSP72 levels decrease rapidly (Senf et al., 2008). Both the upregulation of HSP72 caused by heat stress and transgenic overexpression of HSP72 in skeletal muscle can protect against disuse-induced muscle atrophy (Smuder et al., 2019; Yoshihara et al., 1985; Ichinoseki-Sekine et al., 1985). Exercise training is also a potent inducer of HSP72, significantly increasing its baseline levels in skeletal muscle (Smuder et al., 1985c; Smuder et al., 2019; Fujino et al., 2009). Experimental evidence from disuse models (e.g., mechanical ventilation (Smuder et al., 1985c; Smuder et al., 2019) and hindlimb suspension (Fujino et al., 2009) has demonstrated that exercise preconditioning effectively counteracts skeletal muscle atrophy through HSP72 upregulation, accompanied by a reduction in oxidative stress levels and the inhibition of the activity of NF-κB and FoxO transcription factors (Smuder et al., 1985c; Smuder et al., 2019). Smuder et al. (2019) found that 14 days of high-intensity aerobic exercise preconditioning (70% maximum oxygen uptake [VO_2max_]) significantly attenuated the MV-induced reduction in the diaphragm fiber cross-sectional area. However, when exercise-induced increases in diaphragmatic HSP72 were blocked, the protective effects of exercise were abolished, further confirming that elevated levels of HSP72 are essential for achieving the full benefit of exercise-induced protection.

#### 2.1.3 SESN2

Members of the SESN family, including SESN1, SESN2, and SESN3, play pivotal roles in maintaining metabolic homeostasis, reducing oxidative stress, and regulating autophagy (Kim et al., 2020). SESN2, in particular, regulates both protein synthesis and autophagy, processes that are essential for protecting skeletal muscle from oxidative damage and atrophy, through its inhibitory effect on the mTOR pathway (Saxton and Sabatini, 2017). Exercise serves as a potent inducer of SESN2, with both aerobic (Wang et al., 2018) and resistance training (Yang et al., 2023) significantly upregulating its expression in active skeletal muscles. Elevated SESN2 levels in skeletal muscle enhance antioxidant capacity and maintain protein homeostasis, thereby protecting against muscle atrophy. Huang et al. (2024) demonstrated that, in C57BL/6J mice, 10 weeks of resistance training was more effective in counteracting 1 week of immobilization-induced muscle atrophy than aerobic or concurrent training. This resistance training upregulates SESN2 expression, which is associated with reduced expression of proteolytic enzymes (e.g., FBXO32 and MuRF1), modulation of the AKT/FoxO pathway, and improved protein homeostasis. In contrast, SESN2-knockout mice exhibited significant functional decline and diminished protection against muscle atrophy, even following resistance training preconditioning. This finding revealed the role of SESN2 in mediating the protective effects of exercise preconditioning against disuse-induced muscle atrophy.

### 2.2 Sarcopenia

**FIGURE 2:**
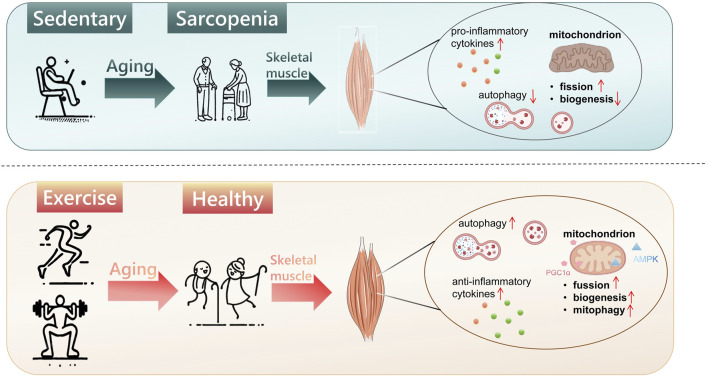
Exercise preconditioning prevents sarcopenia. Aging exacerbates mitochondrial dysfunction, characterized by impaired fusion and reduced biogenesis; suppresses autophagy, and heightens pro-inflammatory cytokine activity, leading to skeletal muscle mass loss and, ultimately, sarcopenia. Exercise preconditioning mitigates these detrimental effects by enhancing mitochondrial function (fusion, biogenesis, and mitophagy), promoting autophagy, and modulating immune responses, thereby preserving muscle mass and function during aging.

With the aging of the world’s population and the increase in life expectancy, age-related health issues, including chronic diseases and functional decline, have emerged as major public health challenges. Sarcopenia, a prevalent age-related condition, is marked by the progressive loss of muscle mass, strength, and physical function, driven predominantly by biological aging and diminished physical activity (Cruz-Jentoft and Sayer, 2019; Chalé-Rush et al., 2010). Reduced physical activity, widely regarded as a pivotal modifiable risk factor, contributes to muscle mass loss and the impairment of muscle function in older adults, primarily by decreasing muscle protein synthesis and disrupting neuromuscular junction integrity (Chalé-Rush et al., 2010). No specific pharmaceutical interventions for sarcopenia are currently available at present, while exercise is the most effective strategy for preventing and mitigating sarcopenia (Rogeri et al., 2021). The 2018 International Clinical Practice Guidelines for Sarcopenia (ICFSR) strongly advocate physical activity, particularly resistance training, as the primary therapeutic strategy for managing sarcopenia (Dent et al., 2018). For instance, pre-aging exercise has been shown to prevent or delay an age-related decline in aerobic capacity by increasing VO_2max_ and muscle capillarization (Gries et al., 1985). A study from the UK National Health and Development Survey (1946 Birth Cohort) found that elevated physical activity levels in middle age can prevent declines in grip strength in later life (ages 60–64). Grip strength, a key metric for assessing sarcopenia, was found to be, on average, 2.11 kg higher in participants in the top third of lifelong physical activity scores compared to those in the bottom third (95% CI: 0.88–3.35) (Dodds et al., 2013). These findings indicate the dual role of exercise preconditioning as both a preventative and therapeutic strategy for sarcopenia, preserving muscle function while mitigating muscle loss (Figure 2).

#### 2.2.1 Mitochondria

##### 2.2.1.1 Mitochondrial dynamics

Mitochondrial dynamics are highly sensitive to fluctuations in nutrient availability and energy demands. Aging disrupts the balance between fusion and fission, reduced expression of key fusion proteins, such as MFN1, MFN2, and OPA1, promoting the fragmentation of mitochondria, which impair muscle function and reduces their metabolic efficiency (Liu D. et al., 2021; Joseph et al., 2013). Exercise has been shown to restore the balance between mitochondrial fusion and fission, primarily by promoting fusion in older individuals, thereby enhances skeletal muscle mitochondrial integrity and network functionality (Kitaoka et al., 2015). For instance, a 4-month exercise intervention in older individuals improved mitochondrial content by significantly increasing OPA1 levels, thereby enhancing aerobic capacity (Arribat et al., 2019). Lifelong exercise confers similar benefits by maintaining the expression of fusion proteins, optimizing mitochondrial dynamics, and protecting against age-related mitochondrial dysfunction, thereby preserving muscle health (Balan et al., 2019). In *Caenorhabditis elegans*, exercise has been demonstrated to delay mitochondrial fragmentation and attenuate the age-related decline in physical fitness. However, these beneficial effects were abolished in worms with knockout mutations of fusion and fission proteins (fzo-1; drp-1 and eat-3; drp-1) (Campos et al., 2023). Similarly, in animal models, long-term exercise has been shown to reduce mitochondrial fragmentation, enhance mitochondrial connectivity, and increase citrate synthase (CS) activity in aged mice. Notably, these effects were absent in PGC-1α-knockout mice (Halling et al., 2017). Consistent with these findings, exercise preconditioning failed to provide protective effects on mitochondrial dynamics and muscle mass in AMPK-knockout worms. Collectively, these observations underscore the critical role of the AMPK/PGC-1α pathway in mediating exercise-induced mitochondrial dynamics and preserving physical fitness (Campos et al., 2023).

##### 2.2.1.2 Mitochondrial biogenesis

Mitochondrial biogenesis is impaired in aging muscle, leading to reductions in both mitochondrial function and number, accompanied by the downregulation of key mitochondrial biogenesis regulators (Zhu et al., 2023; Foreman et al., 2021). Transcriptomic analysis of muscle biopsies from individuals with sarcopenia revealed reduced mitochondrial numbers, decreased respiratory complex activity, and low PGC-1α/ERRα signaling (Migliavacca et al., 2019). In SAMP8 mice (a sarcopenia model), the expression of PGC-1α, NRF1, and TFAM is reduced in skeletal muscle, which contributes to impaired mitochondrial biogenesis and decreased muscle endurance (Liu et al., 2020). Endurance training stimulates mitochondrial biogenesis while activating key signaling pathways, such as those involving PGC-1α and AMPK, thereby improving mitochondrial function and antioxidant capacity. For instance, long-term exercise has been shown to enhance aerobic capacity, mitochondrial content (mtDNA/nDNA ratio), and CS activity in older individuals (Gries et al., 1985; Balan et al., 2019), effects that are largely driven by the upregulation of the expression of mitochondrial biogenesis-related proteins (PGC-1α, TFAM, and NRF1) (Moore et al., 2019). In aging mice, lifelong exercise further enhances CS activity, mtDNA content (Muhammad and Allam, 2018), and the expression of key proteins involved in oxidative phosphorylation and angiogenesis, such as Cyt c, VEGF, and PDHE1α. In contrast, PGC-1α-knockout mice do not exhibit these exercise-induced improvements, suggesting the indispensable role of PGC-1α in regulating mitochondrial biogenesis and mitigating sarcopenia progression (Ringholm et al., 2013). Leick et al. (2010) found that following exercise training, PGC-1α-knockout mice (2–13 months of age) exhibited a 50%–150% reduction in CS activity, as well as reduced Cyt c and SOD2 expression relative to their wild-type counterparts. A separate study applied 8 months of high-intensity interval training (HIIT) to mice (from 18 to 26 months). The authors found that the levels of mitochondrial biogenesis proteins (PGC-1α, Succinate Dehydrogenase Complex Subunit A, Sirtuin 3) and antioxidant enzymes were significantly increased in the soleus muscle, which enhanced mitochondrial function. HIIT also activated the AMPK pathway, promoted mitochondrial super-complex formation, and reduced oxidative stress by limiting ROS production (Han et al., 2022).

##### 2.2.1.3 Mitophagy

Mitophagy, a selective form of autophagy, is critical for the identification and degradation of damaged or dysfunctional mitochondria, thereby contributing to the maintenance of cellular homeostasis and mitochondrial quality (Springer and Macleod, 2016). Although aging is associated with the accumulation of damaged mitochondria, its impact on skeletal muscle mitophagy remains unclear, with studies reporting conflicting results. Some studies demonstrated that mitophagy increases as a compensatory response to damaged mitochondria, potentially reducing mitochondrial content in aging muscle (Hood et al., 2019; Zhang et al., 2020). Others have reported a decline in the levels of key mitophagy markers, such as PINK1 and Parkin, resulting in the acceleration of mitochondrial dysfunction (Liang et al., 2021; Zeng et al., 2020). These discrepancies may be due to differences in experimental methods or the compensatory nature of mitophagy in response to oxidative stress, rather than its role in effectively eliminating damaged mitochondria (Wang et al., 2023). Research suggests that prolonged endurance training increases basal mitophagy levels, which optimizes mitochondrial function, enhances muscle metabolic capacity, and ultimately improves muscular health (Hood et al., 2019). Balan et al. (2019) found that lifelong training enhanced the mtDNA/nDNA ratio, CS activity, and aerobic capacity and increased the expression of mitophagy regulators such as beclin-1, although total Parkin protein levels remained unchanged. Animal studies further support the role of exercise in enhancing mitochondria function. For instance, Zeng et al. (Liang et al., 2021) showed that 12 weeks of various training modalities (aerobic, resistance, concurrent, and voluntary running) increased the expression of the mitophagy protein PINK1, the autophagy regulator beclin-1, Bcl-2, and BAX, as well as the LC3-II/LC3-I ratio, in skeletal muscle, which delayed age-related muscle mass loss. Additionally, 14 months of pre-aging exercise intervention enhanced gastrocnemius muscle mass and cross-sectional area in aged rats, concomitant with upregulated expression of mitophagy regulators (PINK1, Parkin), and AMPK/PGC-1α pathway activation. This adaptive response was further evidenced by elevated citrate synthase (CS) activity, collectively counteracting mitochondrial dysfunction and ubiquitin-proteasome system dysregulation (Zeng et al., 2020). In summary, exercise preconditioning enhances mitophagy by upregulating key regulatory pathways, thereby mitigating sarcopenia.

#### 2.2.2 Autophagy

Autophagy is a conserved catabolic process that maintains cellular homeostasis *via* the degradation of damaged organelles and proteins. In aging skeletal muscle, autophagy-related genes (ATGs) exhibit significant changes that contribute to the development of sarcopenia, a condition characterized by muscle atrophy and loss of strength. As aging progresses, the expression of key autophagy genes such as Atg5, Atg7, and LC3 declines, leading to impaired autophagic flux and reduced protein degradation capacity. This decline disrupts protein homeostasis, resulting in the accumulation of damaged proteins and organelles, which exacerbates muscle atrophy (Xie et al., 2023). Evidence indicates that appropriate exercise interventions can reactivate autophagic pathways, restore muscle function, and slow muscle atrophy. Gao et al. (2020) demonstrated that long-term or lifelong aerobic exercise reduced oxidative stress, and preserved protein homeostasis, thereby mitigating the loss of muscle mass and function in aged rats. These benefits were elicited *via* the enhancement of autophagic activity mediated through the BDNF/AKT signaling pathway. Wang et al. (2022) demonstrated that long-term aerobic and resistance exercise resulted in the upregulation of the expression of autophagy-related proteins in aging skeletal muscle, triggering autophagy and delaying muscle mass loss. Furthermore, different types and intensities of exercise (treadmill running, wheel running, resistance training) exert distinct effects on the expression of autophagy-related protein. White et al. (2016) found that a 34-week voluntary resistance wheel-running program reversed age-related declines in the LC3‐II/LC3‐I ratio and mitochondrial dysfunction and increased muscle mass. Similar results were observed in human studies, where exercise preconditioning was observed to upregulate the levels of autophagy-related proteins in muscle of older adults, restoring aerobic capacity and improving muscle function (Balan et al., 2019). Zeng et al. (2020) proposed that the benefits of exercise preconditioning are mediated through the AMPK/PGC-1α pathway, which enhances autophagic activity, thus supporting muscle health during aging. Overall, exercise preconditioning effectively reactivates autophagic pathways and preserves muscle mass and function in aging skeletal muscle.

#### 2.2.3 Inflammatory response

The inflammatory response is a protective immune mechanism triggered by injury, infection, or harmful stimuli, involving coordinated immune cell activation and cytokine release to eliminate pathogens, repair tissue damage, and restore systemic homeostasis (Santoro et al., 2021). With aging, a phenomenon known as inflammaging emerges, is characterized by persistent activation of the innate immune system, particularly macrophages, and a diminished adaptive immune response (Franceschi et al., 2018). Exercise mitigates age-induced muscle loss by modulating inflammatory responses (Lo et al., 2020). Li et al. (2018) reported that both 8 weeks of moderate-intensity continuous training or HIIT modalities reduced age-related muscle mass decline by decreasing inflammation in aged rats. HIIT was more effective in reversing age-related changes in body composition, and exercise performance, significantly increasing those of anti-inflammatory cytokine Interleukin-10 (IL-10), thereby better preventing muscle atrophy. Master athletes (53.1 ± 8.8 years, ≥20 years training) exhibited higher VO_2max_, lower Tumor Necrosis Factor-α/IL-10 ratios, and elevated anti-inflammatory cytokines (Interleukin-1 Receptor Antagonist, Interleukin-4, IL-10) compared to age-matched untrained peers—with both resting and post-exercise profiles resembling young adults (∼30 years) (Minuzzi et al., 2019). Similarly, lifelong exercisers (74 ± 1 year) exhibited superior VO_2max_ and higher anti-inflammatory mediators (IL-10, Transforming Growth Factor-β, and the PGE2 receptor EP4) alongside lower serum Interleukin-6 (IL-6) compared to age-matched untrained peers, with attenuated post-exercise IL-6 responses mirroring youth exercisers, indicative of reduced systemic inflammation (Lavin et al., 1985).

#### 2.2.4 Myokines

Myokines are cytokines and chemokines secreted by skeletal muscle during contraction—play a vital role in regulating muscle growth, repair, and metabolism. However, certain myokines exhibit age-dependent expression changes that contribute to the development of sarcopenia. For instance, key myokines such as Insulin-like Growth Factor 1 (Ferrari et al., 2021), Brain-Derived Neurotrophic Factor (BDNF) (Coelho et al., 2013), Interleukin-7 (Passtoors et al., 2015), Interleukin-15 (Alcalde-Estévez et al., 2025), irisin (Zhang et al., 2022), and apelin (Vinel et al., 2018), which are essential for maintaining muscle mass and homeostasis, are downregulated with advancing age. Conversely, myostatin (Skrzypczak et al., 2021; Choi et al., 2020), a potent negative regulator of muscle mass, is upregulated in aging muscle, thereby inhibiting protein synthesis and promoting muscle atrophy. Additionally, IL-6, a myokine with both pro-inflammatory and metabolic regulatory functions, exhibits a “double-edged sword” effect on skeletal muscle. These age-related changes in myokine profiles disrupt muscle homeostasis and contribute to the decline in muscle function and mass associated with aging. Regular exercise can reverse this trend by stimulating the release of key myokines such as Insulin-like Growth Factor 1, BDNF, Interleukin-7, Interleukin-15, irisin, and apelin, which promote muscle growth, repair, and metabolic homeostasis, while also downregulating myostatin expression to inhibit muscle atrophy (Bilski et al., 2022; Barbalho et al., 2020). Physical activity, particularly moderate-intensity exercise, significantly increases peripheral BDNF levels in elderly women, promoting neuroprotection and muscle health (Coelho et al., 2013). This effect is consistently observed across non-frail and pre-frail cohorts following physical training interventions (Coelho et al., 2012). These findings highlight the potential of exercise-induced myokine modulation to counteract sarcopenia and warrant further investigation into optimizing exercise protocols.

### 2.3 Cachexia

Cancer cachexia is a systemic metabolic disorder characterized by an involuntary, progressive loss of body mass due to reduced anabolic processes and increased catabolic activity, with skeletal muscle serving as the primary site of protein loss (Dolly et al., 2020; Aquila et al., 2020). Exercise interventions have proven effective in counteracting cachexia-induced muscle atrophy, improving quality of life (Tanaka et al., 2020). Jee et al. (2016) demonstrated that 4 weeks of aerobic exercise preconditioning effectively mitigated cachexia-induced muscle atrophy in mice, with high-intensity exercise yielding superior results in increasing muscle mass and reducing inflammation. Additionally, moderate- and low-intensity exercise preconditioning also provided protection by alleviating capillary regression, mitochondrial dysfunction, and muscle hypoxia, as well as by modulating protein synthesis and degradation pathways (Tanaka et al., 2020; Tanaka et al., 2019). Furthermore, Tanaka et al. (2020) showed that exercise preconditioning provided greater protection to fast-twitch muscles (e.g., plantaris) than to slow-twitch muscles (e.g., soleus), as evidenced by lower muscle mass loss, enhanced mitochondrial function, and reduced oxidative stress. These findings suggest that targeted exercise interventions may be more effective at mitigating cachexia-induced muscle atrophy and functional impairments. In addition to aerobic exercise, concurrent training preconditioning has also been shown to counteract cachexia-induced muscle atrophy. Ranjbar et al. (2019) demonstrated that 6 weeks of concurrent training, significantly enhanced muscle mass, grip strength, and the expression of autophagy markers such as the LC3B-I/II ratio and p62, while also improving mitochondrial function. Although no significant changes were observed in the protein levels of PGC-1α, the activity of Succinate Dehydrogenase was increased, suggesting that mitochondrial function had been partially restored. However, 11 weeks of resistance training preconditioning (3 days/week, starting at 50% of body weight, with 10% weekly increases) did not outperform aerobic exercise in mitigating cachexia-induced muscle atrophy (Khamoui et al., 2016). Taken together, exercise preconditioning, especially aerobic and high-intensity training, represents a promising strategy for counteracting cancer cachexia-induced muscle atrophy and enhancing overall muscle function. Future research should focus on optimizing exercise protocols and exploring their potential in diverse patient populations.

### 2.4 Pharmacological intervention

Exercise preconditioning effectively counteracts drug-induced skeletal muscle atrophy, particularly that associated with agents such as DOX, which causes oxidative stress and proteolytic damage, and DEX, a glucocorticoid that accelerates muscle protein degradation. For instance, 10 days of moderate-intensity aerobic exercise significantly protected rat soleus muscles from DOX-induced damage by enhancing the cellular stress response (upregulating heat shock protein expression), inhibiting DOX-triggered proteolytic systems (e.g., calpain or caspase-3 activation), and reducing oxidative damage and excessive autophagy. These combined effects preserve skeletal muscle function at multiple levels (Kavazis et al., 1985; Smuder et al., 1985a; Smuder et al., 1985b). Resistance training (Macedo et al., 2014; Krug et al., 2016) effectively mitigates DEX-induced muscle atrophy. For example, in a 10-day DEX administration model, resistance training significantly enhanced maximal voluntary carrying capacity (MVCC) and muscle mass by increasing muscle protein synthesis and reducing protein degradation. Both low-intensity (60% MVCC) (Macedo et al., 2014) and high-intensity (80% MVCC) resistance training (Krug et al., 2016) significantly enhanced the MVCC and flexor hallucis longus muscle mass. Interestingly, only low-intensity training increased the mass of the TA, whereas neither modality improved that of the soleus muscle. This may be because the flexor hallucis longus muscle is the primary muscle activated during ladder climbing, whereas the soleus and TA play a lesser role (Macedo et al., 2014; Krug et al., 2016). Yang et al. (2023) demonstrated that 11 weeks of a ladder-climbing protocol (75% MVCC) significantly increased grip strength, MVCC, and muscle mass through SESN2 upregulation. The overexpression of SESN2 can alleviate DEX-induced muscle cell atrophy by inhibiting the FoxO3a and MSTN/Smad signaling pathways, thereby suppressing the activation of the ubiquitin-proteasome system and preventing excessive protein degradation. Unlike resistance training, reports on the effects of aerobic exercise preconditioning on DEX-induced muscle atrophy have been inconsistent. Barel et al. (2010) found that 8 weeks of moderate-intensity aerobic exercise (60% of maximal speed, 1 h/day, 5 days/week) significantly prolonged the time to exhaustion and alleviated DEX-induced atrophy in the extensor digitorum longus muscle of mice. In contrast, Constantino et al. (2017) subjected rats to 60 days of moderate-intensity aerobic training (50%–60% of maximal capacity, 1 h/day, 5 days/week), and observed no significant change in the mass of the soleus, TA, or lumbar muscles following DEX administration. This discrepancy may be attributed to variations in muscle group involvement and species differences, suggesting that aerobic exercise preconditioning may exert muscle-specific effects potentially associated with differences in muscle fiber type composition and activity levels during exercise.

## 3 Discussion

Exercise preconditioning counteracts skeletal muscle atrophy through a variety of mechanisms, including improving mitochondrial function, upregulating cytoprotective proteins, and modulating the inflammatory response, myokine secretion, and autophagy. However, current exercise regimens predominantly focus on standardized endurance or resistance protocols. Future studies should systematically explore diversified strategies—such as eccentric loading regimens and multimodal approaches combining aerobic and anaerobic stimuli—to identify optimal combinations of intensity, frequency, and modality that synergistically target multiple atrophy pathways. While exercise-based interventions hold therapeutic promise for muscle preservation, their clinical applicability is significantly limited under pathophysiological conditions characterized by prolonged immobilization or advanced cachexia, where physical activity is impractical. In such contexts, alternative therapeutic modalities—such as neuromuscular electrical stimulation (NMES), robotic exoskeleton-assisted rehabilitation, and precision-targeted acupuncture protocols—emerge as viable options for preserving musculoskeletal structure and function. Mitochondrial dysfunction is a key driver of muscle atrophy, yet the precise mechanisms remain to be fully elucidated. Future research should focus on how impairments in mitochondrial function and bioenergetic capacity contribute to muscle loss, and determine the extent to which exercise preconditioning can counteract these deleterious effects. The concept of muscle memory and its role in exercise preconditioning’s ability to mitigate muscle atrophy remains largely unexplored. Future research should focus on defining muscle memory in this context and elucidating the mechanisms through which exercise preconditioning prevents muscle atrophy *via* the establishment of muscle memory. Addressing these knowledge gaps will not only deepen our understanding of the protective benefits of exercise preconditioning but also support the development of more effective, personalized strategies for preserving skeletal muscle health across diverse populations.

## Abbreviations

AMPK, Adenosine Monophosphate-Activated Protein Kinase; BDNF, Brain-Derived Neurotrophic Factor; CS, citrate synthase activity; DEX, dexamethasone; DOX, doxorubicin; ERRα, estrogen-related receptor alpha; HIIT, high-intensity interval training; HSP72, Heat Shock Protein 72; ICFSR, International Clinical Practice Guidelines for Sarcopenia; IL-6, Interleukin-6; IL-10, Interleukin-10; mtDNA, mitochondrial DNA; MV, mechanical ventilation; MVCC, maximal voluntary carrying capacity; NRF1/2, nuclear respiratory factors 1/2; PGC-1α, proliferator-activated receptor γcoactivator-1α; ROS, reactive oxygen species; SOD, superoxide dismutase; TFAM, mitochondrial transcription factor A; VO_2max_, Maximum Oxygen Uptake.
